# Treatment of Dental Caries, Complicated with Affections of the Pulp and Peridental Membrane

**Published:** 1851-04

**Authors:** Robert Arthur


### AMERICAN JOURNAL

OF

### DENTAL SCIENCE.

Vol. I. NEW SERIES—APRIL, 1851. No. 3.

### ARTICLE I.

*Treatment of Dental Caries, complicated with Affections of the Pulp and Peridental Membrane.* By ROBERT ARTHUR, D. D. S.

NO. I.

HAVING, for some years past, directed my attention, with interest, to the subjects which give a title to this paper, I now propose to present to the profession the results of my experience in relation to them, and also what I have gathered in my intercourse with other members of the profession.

The very satisfactory results which, in my own hands, have followed the practice of the operation of the entire extirpation of the dental pulp, and what I know to be the results of this practice in the hands of several of my friends, satisfy me that great good may be accomplished if the general attention of the profession can be seriously directed to this subject. The denunciations of this operation which, from time to time, have appeared in various places, have satisfied me that, when attempted, it could not have been performed in the thorough manner which I know is essential to success. As success has followed the practice which I have adopted, (and which is not by any means original with me, or peculiar to myself,) I have felt it a duty to state, in full detail, what that practice is, so that it may be fairly and faithfully tried by all who think the objects to be gained worth the trial. If it is fairly and faithfully tried, I cannot see how the results can be different from what I and others have found them to be; and if these are obtained, I am sure any operator who adopts the practice, will be well repaid for his pains.

The general outlines of this practice have already been drawn, both in an editorial article which appeared in vol. vii of the "Journal," by Dr. Westcott, in an account of a visit to Dr. Maynard of this city; and in a series of excellent articles on the subject, by Dr. J. D. White, of Philadelphia, first published in the "Dental News Letter," of that city, and afterwards copied into the "Journal." But, in order that this operation should be attempted with probability of success, by those who have never before practiced it, I am satisfied that more should be told in detail, than what is stated in those articles. This is what I propose to do.

When I first thought of publishing my views upon this subject, it was my intention to confine myself to that branch of it to which I have alluded above. But, on considering the matter more attentively, I found so closely connected with this subject, several others, that I enlarged the original scope of my design, so as to embrace the treatment of the teeth after the pulp may have become in any manner involved, or in danger of being involved, in disease, or in the progress of necessary preservative operations. And as disorders of the peridental membrane so often accompany or are occasioned by treatment of the pulp, some examination of these disorders seemed to come naturally into this place.

It is generally an ungracious thing to make apologies in advance, for conscious imperfections of a work, and it may, perhaps, be said by some, that a writer ought to get rid of imperfections in what he ventures to publish. This, however, can never entirely be. And when a man is satisfied that he is able to say something which may either be useful in itself, or be likely to suggest what may be productive of good, he ought to say it without delay. I am quite as well aware as any of my readers can ever become, of what will be found imperfect in the following treatise, and I can only hope that they will be stimulated to endeavor to supply all defects which they may discover in it.

As this treatise is intended, for the present at least, for members of the dental profession, who are supposed to be well acquainted with the anatomy of the teeth, I have determined to abandon my original intention of devoting a portion of it to a minute description of the dental pulp, and the cavities in which it is contained, in the various classes of the teeth. If, however, this effort proves to possess any value, and is favorably received, I may, at some future time, add these preliminary descriptions, and also the results of a number of investigations, upon which I am now engaged, in relation to the general subject, which cannot but prove useful, at least to the student of dental surgery, if not to the profession at large.

The preservation of the teeth, after that portion which is now generally designated the pulp, has been deprived of its bony covering, by disease or otherwise, has long been a desideratum, and has, on all hands, been acknowledged an object difficult of accomplishment. Many eminent practitioners of dental surgery, till within a very late period, have declared their belief, that any operation having this end in view, except in a very limited number of cases, would result to the injury of the patient upon whom it is practiced, and have accordingly denounced it in severe terms. A change, however, I am glad to perceive, is gradually taking place in this respect, and saves the necessity of meeting objections which have been urged against it on a variety of grounds—objections which it would be an easy matter to set aside.

Several methods have been employed to accomplish the object alluded to. These shall be briefly examined.

The French practitioners, who have, for many years, given much attention to the subject, advised, generally, the entire extirpation of the pulp, and relied, principally, upon the actual cautery to effect this object. Many careful descriptions of the instruments for the purpose, and the manner in which they should be employed, are given by them. This operation, with them, however, was confined to the superior incisor and the inferior bicuspid teeth, which, they allege, are the only teeth in which the pulp, in its whole length, can be reached with the caurerizing instrument.

It was directed, after opening the internal cavity, so as to remove all obstruction, to heat the instrument, and thrust it quickly into the pulp, and to repeat this operation as long as any sensibility remained. Several days or weeks were then allowed to elapse, for the dissipation of any inflammation which might ensue, when the tooth was filled in the ordinary manner.

Fauchard, (vol. i, p. 175,) in a chapter on the treatment of painful teeth, describes an operation, which he calls trepanning the teeth. It is simply piercing the pulp with a fine needle, from which the temper has been taken out. After this has been done, for the purpose, he says, of giving egress to the humors which may be retained in the dental cavity, the tooth is to be left untouched for several weeks. The external cavity, he says, is to be filled with cotton, to prevent decay in the meantime, but care must be taken to avoid packing this so tightly as to prevent the escape of any matter which may be discharged from the dental cavity. Indeed, he sometimes found it necessary to remove, several times, the final metallic filling, to relieve pain which followed the operation from this cause. With the incisor and canine teeth, he alleges, he was generally successful in relieving the pain; and he advises that the operation should be confined to these. In cases where pain is not entirely relieved by this operation, he refers it to the peridental membrane, and notices, too, the fact, that pain sometimes continues even after the extraction of such teeth.

It is proposed, by Hunter, to extract teeth after the nerves have become exposed, boil them with a view to render them perfectly clean, and then return them to the sockets; after which, he says, as they are now dead, they cannot suffer any further from disease, but can only be acted upon chemically or mechanically. This practice he recommends in grinders only. No comment upon this practice is necessary at the present day. If the patient will not suffer the extraction of a tooth affected in this manner, (an operation to which the author alluded to gives preference,) he must have the nerve burned, and this, in order to be successful, must be done to the apex of the fang, which is not always possible. Any one of the concentrated acids is capable of destroying the soft parts of a tooth, if brought into contact with them. To the lower teeth these substances, he says, may be readily and conveniently applied, as they will pass down into the fang by their own gravity; but, for the same reason, they are not readily applicable to the upper teeth. For this purpose, caustic alkali is recommended, placed upon a dossil of lint, and pushed up into the dental canal.—(Hunter, Am. ed., part ii, pp. 7, 8.)

It has also been recommended to effect the destruction of the pulp, by the partial luxation of the painful tooth. The operation consisted in removing the tooth so far from the socket as to break up its connection with the jaw, and then immediately return it to its place.

The whole of the above class of agents have reference to the entire destruction of the dental pulp after it may have become exposed. The authorities for these methods of practice might be very much increased; but as they have deservedly gone almost, if not entirely, out of use, it is, of course, unnecessary.

Mr. Thomas Bell, (p. 151,) after noticing several of the methods of destroying the nerve mentioned above, with, it seems to me, unanswerable objections, deprecates the practice as painful and inefficient for the intended purpose. He does not place reliance upon any operation for the preservation of a tooth after the pulp has once become extensively exposed, but believes that all attempts of this kind will result to the injury of the patient. When, however, the pulp is very slightly exposed, he advises the use of agents which will remove its sensibility, or produce its absorption, "so as to render it capable of receiving the stopping without any pain or inconvenience." For this purpose, he made use of alcohol, spirits of camphor, solution of nitrate of silver, &c., kept continually, for some time, in contact with the slightly exposed pulp. "It is," he says, "not easily determined, nor is it of much importance, in what way these applications produce the effect—whether by occasioning the actual absorption of that part of the membrane to which they are applied, or by gradually wearing out, as it were, its sensibility; it is sufficient, that experience proves them to be efficacious. I have generally preferred camphorated spirits or alcohol, as the nitrate of silver requires more caution in its management, and could not, perhaps, with safety, be left to the patient's own care. The liquid should be applied at least three or four times in the day, on a small bit of lint or cotton, the cavity being rendered perfectly dry, and its use should be continued until considerable pressure no longer occasions pain."—Op. cit. 154.

Koecker, (Am. ed., p. 226,) in taking up this subject, after having denounced the practice of destroying the nerves of teeth in the manner commonly adopted at the time he wrote, asserts that the only rational manner in which a tooth can be treated after the exposure of the pulp, is the restoration to, and preservation of the health of this important part of the tooth. For this purpose, he adopted local treatment, to obtain the following results:

"1. To put a stop to the caries, and consequently to prevent the irritation upon the lining membrane of the tooth.

"2. To suppress the hemorrhage, and to cure the membrane if wounded.

"3. To protect the membrane, artificially, against the action of all foreign agents."

If the cavity of decay could be properly prepared without wounding the pulp, Dr. Koecker at once filled the cavity in the usual manner. But if the pulp was wounded, he arrested the hemorrhage by the actual cautery, which he applied by means of an iron wire, filed and bent, so as to adapt it perfectly to the form of the part of the pulp exposed; this he applied repeatedly to the part, taking care to avoid touching the adjacent bone. After having done this, he wiped out the cavity—observing great caution not again to wound the pulp: allowed sufficient time to elapse for the original wound of the pulp to heal, when he proceeded to effect the third object proposed by him, the protection of this portion of the tooth against foreign agents. This he accomplished by laying over the exposed pulp a piece of thin leaf lead, and then filling up the cavity with gold, in the usual manner. After the completion of the operaton, Dr. K. advises the patient to avoid any causes which will be likely to excite inflammation in the surrounding parts, and takes the proper means of subduing it on its first appearance. Dr. K. asserts that five out of six teeth may be preserved alive by this treatment, and mentions a case in which he succeeded perfectly with six, viz. one upper incisor, one cuspid, two bicuspids, one under bicuspid, and one molar.

I believe I have now presented the substance at least of what is on record, which has any bearing upon the treatment of the dental pulp, previously to the introduction of the arsenious acid as an agent for the purpose of effecting the entire destruction of this portion of the tooth.

It will be seen that little was done, so far at least as we may judge from what is on record, towards the performance of a thorough operation for the removal of the dental pulp, and the subsequent treatment of the tooth. The French writers recommended the removal of the pulp, either with an instrument, without any preparatory steps, or to destroy the sensibility, previously to doing this, by the actual cautery. Much was said about the manner of applying the cautery, but beyond this, the directions given for the subsequent treatment of the tooth, are very meagre indeed.

English writers generally condemned the operation, as will have been seen, and preferred making an effort to save the pulp alive.

The operation now practiced in this country, for the treatment of the teeth, after the pulp may have become uncovered, is entirely to remove this part of the tooth, as far up into the root as it can be reached by means of a delicate instrument, and after this is effectually done, to fill the place occupied by the pulp, up to the apex of the root, with gold.

It is difficult now to say, with certainty, who deserves the credit of having originated this operation. Dr. E. Baker has made, as far as I am aware, the first publication in relation to the subject,* and after describing briefly the operation, states that the late Dr. Hudson, of Philadelphia, was in the habit, thirty years before that time, (1839,) of treating the front teeth in this manner, and with great success.

The operation for the entire extirpation of the pulp, and the complete filling of the fang, divides itself into two classes—that which confines it to the incisor and bicuspid teeth, and that which embraces all classes of the teeth. From evidence presented, it appears that the French practitioners, many years ago, were in the habit of removing the body of the pulp; but nothing has been left on record which will justify the belief that they attempted to practice the thorough operation of filling the fangs. Dr. Maynard, who appears to have taken great pains, during a late visit to Europe, to ascertain the exact condition of dental surgery there, states that he did not meet with a single individual, in England or on the continent, who had practiced the operation, and that he introduced it into St. Petersburg.

Dr. Baker, in the article above alluded to, states that the late Dr. Hudson, of Philadelphia, was in the habit of removing the pulp from the canal in the roots of the teeth, and filling carefully with gold; but he does not say this operation was performed upon the molar teeth. For a long time, whilst it has been admitted by some of the most decided opponents of the practice, that the operation might be allowable when confined to the incisor teeth, it was objected to, without qualification, when practiced upon the molar teeth. Now the operation, as applied to the molar teeth, I consider the most important feature of the treatment, for these teeth are really, in many cases, more valuable to the patient than the incisor teeth, which can be more easily replaced when lost.

I have carefully inquired into the origin of this operation upon the molar teeth. My attention was first called to it by Dr.

* See Am. Jour. Dental Science, vol. i, p. 171.

J. D. White, of Philadelphia, who stated to me, it is now my impression, that he practiced it about ten years ago. Dr. E. Townsend, of Philadelphia, (see Journal for October, 1850,) states that he began to practice the operation in 1840, and has since performed it in many cases, but does not state whether he confined his operations to the incisor teeth, or practiced it without regard to the class. The first publication on the subject of performing this operation upon the molar teeth, as far as I am aware, was made by Dr. Dunning, in a paper on "Practical Dentistry," read before the sixth annual meeting of the American Society of Dental Surgeons, and published in the Journal of 1845. Dr. D., in this paper, objects to the use of arsenic for destroying the vitality of the pulp, and advises its removal with an instrument, without any preparatory treatment. After practicing it, more or less imperfectly, for several years, I took up my residence in Washington City. Dr. Maynard then described to me his most thorough manner of operating in these cases, and I adopted improvements he suggested, with great advantage. In conversation with him, he says he filled a molar tooth in this manner, as nearly as he can recollect, about thirteen years ago. He states, however, that the operation is not original with him; that it was first suggested to him by Dr. Cherry, of Alexandria, and that it is his impression that the Drs. Tucker, of Boston, practiced it long before he did. Of one thing, however, from a variety of evidence, convincing to me, I am satisfied that he was among the first, if not the first, who performed this delicate operation in the thorough manner in which it is now generally done to insure success. And I am satisfied that the present credit the operation has obtained in the profession, is, in a great measure, due to him.

Up to the year 1836, as far as I can learn, the operation of destroying and removing the dental pulp, was limited, in this country, to the incisor teeth, and was practiced by a few individuals only. At that time, the arsenious acid, as an agent for the destruction of the vitality of the pulp, previously to its removal, was introduced to the profession by Dr. S. Spooner, in a popular treatise, entitled "A Guide to Sound Teeth."

The use of this agent removed one of the most serious objections to the operation—its extreme painfulness. The surprising effects, and the uniform certainty with which they followed the application of this substance, induced, at once, its almost universal employment for the purpose of destroying the vitality of the pulp. It now became a common practice, not only to destroy the pulp of the front teeth, but of all the teeth. When a patient presented himself, to have a painful tooth removed, he was told that he could be certainly relieved of pain, without the loss of the tooth, which, probably of great value to him, could be saved and rendered useful. The tortured patient, glad to escape the terrible operation of extraction, readily consented. The arsenic was applied. If the pain did not immediately cease, in the course of a few hours at least he would be relieved. The next day, on coming to his dentist, he finds, to his great amazement, that the tooth, which the day before was so exceedingly sensitive that the mere contact of the air rendered him almost frantic, is now perfectly insensible. He can scarcely believe the evidence of his senses. The tooth is at once filled, and he leaves the office, loading his benefactor with thanks, and paying the fee for the operation, with expressions of gratitude. The operator was delighted that he could, with so much certainty and ease, accomplish such an important object.

It may be readily supposed that results such as these, brought it at once into general use. With the better class of practitioners, however, it was soon discarded, for, although the first effects were so satisfactory, it was soon found that bad consequences followed, which, with almost invariable certainty, made the extraction of the tooth, so treated, at last necessary. In the course of a few days, or sometimes weeks, inflammation of the peridental membrane set in; the whilom comfortable and useful tooth became at first tender to pressure, and apparently a little longer than its neighbors; in a few days more, came an attack of pain more serious than that which a few weeks before was so successfully relieved; the gum near the tooth enlarges, the pain becomes intense. The sufferer either goes at once and has the offending member extracted, or poultices his face to hasten suppuration. After suffering excruciating pain for a time longer, relief came in the shape of an alveolar abscess; an ulcer is formed, and a continuous unpleasant discharge is established. In some cases, in the lower jaw, a fistulous opening is formed in the cheek, and when the cause of all this is finally removed, an unsightly scar is left to disfigure the face for life.

That these were the common results which followed the use of arsenic after its first introduction, is well known, and it is not at all surprising that its use should have been discouraged. In my own case, after using arsenic in this manner for a short time, I threw it aside as injurious.

After a time, however when the use of arsenic for the extirpation of the pulp was generally discontinued amongst the better class of practitioners, it was understood that several members of the profession, some of high repute, were using it habitually, applying it to any class of the teeth, and with alleged success. This brought forth a number of violent attacks upon the practice, and it was denounced as the worst kind of malpractice. No defence of the practice was made, rather from indifference to the attack—a feeling that it was uncourteous—and from a desire, on my own part at least, to let the matter take its own course, till time enough had elapsed to enable me, if no one else would do so, to present the practice in such a light that it would command the attention at least of the profession. But I now find myself forestalled, by a change which appears lately to have taken place in the views of some, who, a few years since, most loudly denounced its use, and who are now disposed, as they should have been long ago, to believe that there was some truth in the positive declarations of practitioners, whose character as men gave them at least a claim to credibility.

But if arsenic is made use of as the destructive agent in this operation, and it has been used improperly heretofore, what is the right manner of using it?

This has already been indicated, as I have said, in an article in the 7th volume of the "Journal," and in a series of excellent articles on the "Treatment of the Dental Pulp," by Dr. White, of Philadelphia. What I propose to do now, is to present a description of the operation, in full detail; the symptoms in the course of treatment which have presented themselves in my hands; the causes of failure, and the cases in which success may be reasonably looked for.

The apparent want of a work specially devoted to this subject, at this time, has induced me to undertake the task. Although the treatment I shall advocate is not yet all that could be desired—still I am convinced, and hope I shall be able to convince others, who are incredulous now, that the subject is one of considerable importance to the community, and to the profession at large.

If a work devoted to this special subject could have been expected from others in the profession, I should most willingly have left it to abler hands. As I have undertaken the task, it shall be my effort to present the subject in a plain, straight-forward way. I shall endeavor to say that which may prove useful, and that only. I shall endeavor carefully to give credit to others, for what they may have done towards the improvement of this valuable practice; and if I should fail, in any instance, to do so, this will arise rather from a want of knowledge of facts, than from intentional neglect.

[To be continued.]

